# Use of validated community-based trachoma trichiasis (TT) case finders to measure the total backlog and detect when elimination threshold is achieved: a TT methodology paper

**DOI:** 10.11604/pamj.2017.27.84.11266

**Published:** 2017-06-05

**Authors:** Jefitha Karimurio, Hillary Rono, Doris Njomo, John Sironka, Catherine Kareko, Michael Gichangi, Ernest Barasa, Alice Mwangi, Kefa Ronald, Francis Kiio

**Affiliations:** 1 Department of Ophthalmology, College of Health Sciences, University of Nairobi, Kenya; 2 Kitale Eye Unit, Kitale, Kenya; 3 Kenya Medical Research Institute, Nairobi, Kenya; 4 Narok Trachoma Elimination Project, Narok, Kenya; 5 Ophthalmic Services Unit, Ministry of Health, Nairobi, Kenya; 6 Operation Eyesight Universal, Nairobi, Kenya; 7 Narok County Government, Narok, Kenya

**Keywords:** Trachoma trichiasis, case finders, TTall, TT15, TT40

## Abstract

**Introduction:**

The World Health Organization recommends TT surveys to be conducted in adults aged 15+ years (TT 15 survey) and certifies elimination of TT as a public health problem when there is less than 1 unknown case per 1,000 people of all ages. There is no standard survey method to accurately confirm this elimination prevalence threshold of 0.1% because rare conditions require large and expensive prevalence survey samples. The aim of this study was to develop an accurate operational research method to measure the total backlog of TT in people of all ages and detect when the elimination threshold is achieved.

**Methods:**

Between July to October 2016, an innovative Community-based, Mapping, Mop-up and Follow-up (CMMF) approach to elimination of TT as a public health problem was developed and tested in Esoit, Siana, Megwara and Naikara sub-locations in Narok County in Kenya. The County had ongoing community-based TT surgical camps and case finders. TT case finders were recruited from existing pool of Community health volunteers (CHV) in the Community Health Strategy Initiative Programme of the Ministry of Health. They were trained, validated and supervised by experienced TT surgeons. A case finder was allocated a population unit with 2 to 3 villages to conduct a de jure pre-survey census, examine all people in the unit and register those with TT (TT all survey). Identified cases were confirmed by TT surgeons prior to surgery. Operated patients were reviewed at 1 day, 2 weeks and 3-6 months. The case finders will also be used to identify and refer new and recurrent cases. People with other eye and medical conditions were treated and referred accordingly. Standardised data collection and computer based data capture tools were used. Case finders kept registers with details of all persons with TT, those operated and those who refused to be operated (refusals). These details informed decision and actions on follow-up and counselling. Progress towards achievement of elimination threshold was assessed by dividing the number of TT cases diagnosed by total population in the population unit multiplied by 1,000.

**Results:**

Narok County Government adopted both the CMMF approach and TT all survey method. All persons in 4,784 households in the four sub-locations were enumerated and examined. The total population projection was 29,548 and pre-survey census 22,912 people. Fifty-three cases of TT were diagnosed. The prevalence was 0.23% and this is equivalent to 2.3 cases per thousand population of all ages. Prior to this study, the project required to operate on at least 30 cases (excess cases) to achieve the elimination threshold of 1 case per 1000 population.

**Conclusion:**

The total backlog of TT was confirmed and the project is now justified to lay claim of having eliminated TT as a public health problem in the study area. TT all method may not be appropriate in settings with high burden of TT. Nomadic migrations affect estimation of population size. Non-trachomatous TT could not be ruled-out.

## Introduction

**Figure 1:**
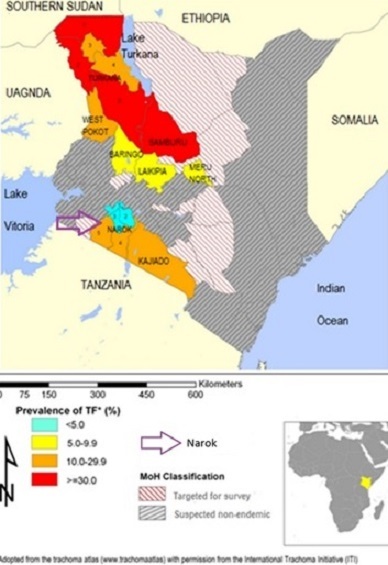
Kenya trachoma inflammation-follicular (TF) map and Narok project (arrow)

**Figure 2:**
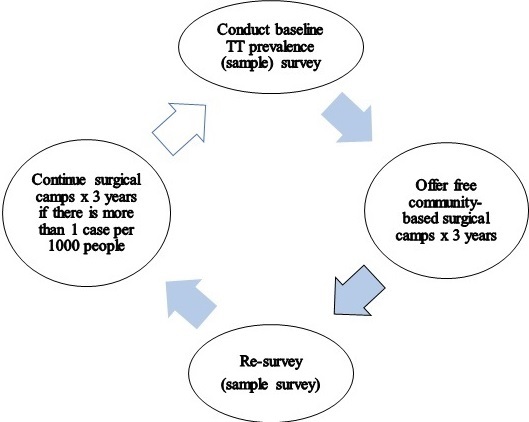
The previous approach for elimination of TT as a public health problem

Trachoma is the leading infectious cause of blindness in the world and TT the monitoring indicator for the potentially blinding stage [1]. TT is a condition where eye lashes turn inwards and scratch on the cornea. It is follows trachomatous conjunctival scaring (TS) caused by repeated infections. Immediate lid surgery is needed because TT is painful and potentially blinding. TS is not used as planning indicator since it does not require intervention and not all cases progress to TT [1, 2]. Kenya is a trachoma-endemic nation in Africa and the disease is localised in the arid areas with poor hygiene and nomadic communities (Figure 1). Narok is one of the 19 community-based trachoma elimination projects in the Kenya Trachoma Elimination Project. Narok project is sponsored by the National Government, County Government, Operation Eyesight Universal and Queen Elizabeth Diamond Jubilee Trust. In 2010, the 158 administrative districts in Kenya were aggregated into 47 Counties. The larger Narok (Narok East, Narok North, Narok South, Narok West) and Transmara (Transmara East and Transmara West) districts were merged to create Narok County. The sub-county administrative units in 2009 national census report were sub-counties, divisions, locations and sub-locations. Villages were not included in census report. The next census is due in 2019. Prior to this operational research, sample surveys were recommended at intervals of 3-5 years to monitor progress towards elimination of TT as a public health problem. Surveys were followed by periodic free community-based eye camps to tackle the projected backlog. Community members were screened and those with TT operated on Figure 2. However, the exact backlog of TT in the community remained unclear as there was no standard prevalence survey method to accurately confirm achievement of the elimination threshold. Surveys to estimate low prevalence thresholds require large samples [3]. Different lower age limits of TT survey participants have been used in an attempt to lower survey samples and cost but they end up lowering precision in estimation of the prevalence and backlog [4]. Between 2004 and 2007 in Kenya, surveys were conducted in people 15+ years old (TT 15) as recommended by the World Health Organization [5–7]. Thereafter, a lower age limit of 40 years (TT 40) was adopted [4]. Lower age limits of 30 and 40 years have been used in the Pacific Islands [8] and Australia [9] respectively. In 2015, the Global Trachoma Mapping Project (GTMP) published a survey method where all persons 1+ years old (TT1) in household selected for active trachoma survey were examined for TT and the main outcome for TT was prevalence in persons 15+ years old. The authors acknowledged low accuracy in estimation of prevalence of TT [10]. In Narok, a baseline prevalence survey was conducted in 2004. The prevalence of TT in persons 15+years old was 2.3(95% CI:1.3%-3.7%) [5]. A district-based trachoma elimination project was launched in 2007 and impact surveys conducted after every 3 years to justify continuation of interventions.

The 2010 and 2014 impact surveys indicated that only 2 southern segments (Figure 1) had remained endemic due to high prevalence of known environmental risk factors [11]. A survey segment was defined as an area with 100,000-200,000 people [12].These population size limits were within the limits of the trachoma intervention unit recommended by the World Health Organization [1]. The 2014 impact survey report indicated that the prevalence of TT in people 40+ years old in the two segments was 5.9%(95% CI: 4.0%-7.7%). Prevalence in women was 3 times higher than in men. The backlog of TT in the two segments was estimated to be 2,323 (95% CI: 1,174-3,048) people. Narok aims to eliminate TT as a public health problem by the year 2020. A Knowledge, Attitudes and Practices (KAP) study conducted in Narok in 2014 revealed that lay people living in endemic areas know TT and have local names for it [12]. Usually TT is easy to diagnose hence formal validation of TT graders is not routinely done in prevalence surveys [13]. However, the project had not utilised non-technical or lay persons to identify TT cases during scientific studies. There is no standardised prevalence survey method available to certify the elimination of TT and all the survey methods described above [4, 5, 10] do not have adequate power to do so. Moreover, eye camps where only the community members who turn up are screened for TT cannot provide accurate data to calculate prevalence in the whole community. Trachoma projects in Kenya have experienced TT case finders who screen for TT cases at the household level but they have not been utilised to conduct prevalence surveys. It is targeted that at least 75 cases should be operated in a single surgical camp for a camp to be considered cost effective but TT cases are getting fewer and thinly spread with time. Consequently, it is becoming increasingly difficult to get optimum cases for ''supply-driven'' camps. Also, project costs are sky rocketing as the elimination threshold is approached. For example, if there is 1 case per 1000 people, then 75,000 people must be screened to get 75 cases. Moreover, it was not clear when to stop the survey-surgical services cycle in Figure 2. It is difficult to plan for elimination of TT without an accurate research method to measure the actual backlog to be tackled and detect when elimination threshold is achieved. Furthermore, there is a need to contain the escalating project costs TT surgical camps. These challenges triggered the need to search for a more effective approach and survey method. Integration of TT surveys into routine project activities was found to be the most feasible option. The broad objective was to develop an operational research method to measure the total backlog of TT and to detect when the elimination threshold is achieved. The specific objectives were to: develop the operational research method; field test the new method in Narok; ascertain whether the elimination threshold has been attained in the studied population The research activities included: two brainstorming meetings and emails communications to develop a new approach; validation of the TT case finders working in the project; field visits to discuss logistics of the research and document processes; de jure census to ascertain the population size of the study area (reference population); house-to-house survey to verify the total backlog and prevalence of TT

## Methods

This operational research was initiated in 2016 as part of a trachoma elimination project in Narok County in Kenya (Figure 1). The methods were designed and field tested by a joint team of trachoma experts from Narok County, Ministry of Health, University of Nairobi, Kenya Medical Research Institute (KEMRI) and Operation Eye Sight Universal (OEU).

### The new approach and survey method

**Figure 3:**
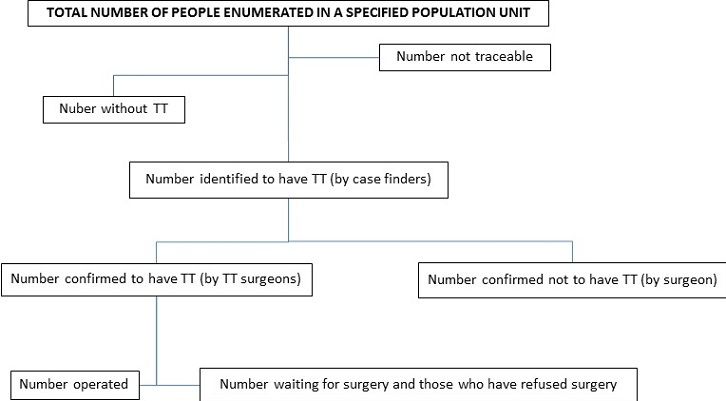
Flow diagram for the new CMMF approach

A community-based, mapping, mop-up and follow-up (CMMF) approach for elimination of TT was designed and validated where case finders were deployed to enumerate, examine all household members for TT and register all TT cases (TT all survey method) in their population units. Narok trachoma project offered free community-based surgical services to all persons with TT. The operated patients were reviewed at 1 day, 2 weeks and 3-6 months. Surveillance system was put in place where the case finders diagnosed and referred recurrent and new (incident) cases. The difference between the previous and new approaches are shown in Table 1 below. The flow diagram for the CMMF approach Figure 3 .

**Table 1:** Comparison of CMMF and previous approaches

Activity	CMMF (new approach)	Previous approach
Baseline survey	- Whole community examined once	- Sample survey
Impact survey	- Not necessary	- Conducted periodically
Community mobilization for to identify cases for surgical camps	- Not needed because all the TT cases in the community are known - Continuous surveillance is integrated in ongoing project activities, relatively cheaper	- Needed because the cases in the community are “unknown” - Stand-alone and it because more expensive as the elimination threshold is approached
TT surgical services	- Continuous and “demand driven”	- Periodic community based surgical camps which are “supply driven”
Follow-up of operated patients	- Needed	- Needed
Surveillance for new TT cases	- Starts at baseline when total backlog is revealed tackled	- No guidelines on how to go about it. The magnitude of unknown cases uncertain

### Target population size and population units

The Kenya National Bureau of Statistics (NBS) provided population projections derived from the national 2009 census report [14]. Besides, the trachoma project team conducted a de jure census where individuals were enumerated in the household where they resided. This triangulation in estimation of population size was necessary because of nomadic migrations. A geographical area with known population size where the project team camped to offer surgical services was referred to as a population unit, irrespective of the population size of the unit. An intervention unit was defined as a geographical area with population size of between s100,000 and 250,000 people as recommended by the World Health Organization [1].

### Case definition of TT

TT was diagnosed if there were one or more eye lashes touching the eye-ball.

### Inclusion criteria

People of all ages (TT all) living in the Narok trachoma elimination project area were eligible to participate in this operational research.

### Exclusion criteria

Non-residents such as traders and tourists were excluded. Local people living in shops in trading centres were also excluded on assumption they would be enumerated in their rural households.

### Recruitment, training and validation

Case finders were recruited from an existing pool of CHV in the MOH Community Health Strategy Initiative (CHS) where Counties are divided into geo-medical units referred to as Community Health Units (CHUs). A CHU comprised of approximately 5,000 people served by 2 Community Health Extension Workers (CHEWs) and 50 Community Health Volunteers (CHVs). A CHV serves 20 households with approximately 100 people [15]. CHVs are not salaried workers. Continuous training and validation of CHVs as TT case finders was done to cater for attrition. The most experienced case finders in Narok were recruited for this study. A further on-the-job training was done where a case finder was paired with a TT surgeon until the case finder was certified as qualified enough to work independently. During the training period both TT surgeon (examiner) and the case finder recorded their findings in separate data collection tools without discussing or disclosing the diagnosis to each other. Ratio of case finders to supervisor is 2:1 to ensure quality supervision. All TT cases identified by case finders were confirmed by TT surgeons.

### Sampling and selection methods

All households were visited (Table 2) and all people in the households enumerated and examined for TT. The project area was divided into small manageable population units with 2 to 3 villages each. A case finder was allocated one population unit. The study commenced in population units of a single sub-location and systematically expanded from sub-location to the next. This was to continue until the whole project area is covered. The community mobilisation, data collection and surgical teams camped in the same sub-location for one week before relocation to the neighbouring sub-location. Household members who were absent during the initial visit were traced, enumerated and examined at their household. Children in local schools were examined at school and data included in their respective households. Persons who were not traceable within the one week period were excluded. These included local people working or studying outside the study area. The one week census period was necessary to mitigate against migrations and operate on diagnosed TT cases. All the population units in Narok project will be systematically surveyed until the total backlog of TT in the project area is known. This publication contains findings for the initial population units.

**Table 2:** Preliminary findings of Narok the TT all survey results

Name of Sub-location	Total Number of households enumerated	Population projection	Total number of people enumerated and examined			Population Coverage (%)*	Total number of people with TT#		
			Male	Female	Total		Diagnosed	Threshold cases+	Excess cases++
Esoit	1257	7445	3642	3792	7434	98.5	13	8	5
Siana A	598	8541	1667	1769	3436	95.6	7	4	3
Siana B	608		2243	1948	3791		14	4	10
Megwara	979	4659	2316	2413	4729	101.5	10	5	5
Naikara A	515	8903	2313	2598	4911	94.7	15	5	10
Naikara B	827		1830	1692	3522		9	4	5
**Total**	**4784**	**29548**	**11698**	**11614**	**22912**	**97.6**	**53**	**23**	**30**

* Population coverage = percentage of the population projection examined

# All persons identified to have TT (by case finders) were confirmed by TT surgeons

+ Threshold cases = maximum number of cases which corresponds to 1 case per thousand examined population

++ Excess cases = Number of cases diagnosed minus threshold cases (which if operated the prevalence would be 1 per 1000 population)

### Examination and data collection methods

Clinical examination was done at the household using x 2.5 dioptres binocular loupes and torches. The following details were recorded in the study data collection form: date of visit/examination, administrative unit, village, telephone contact, age, sex, diagnosis and eye with TT. Certified TT surgeon confirmed all cases identified by case finders. Case finders kept registers with details of all patients with TT, those operated and refusals in their population unit. These details were used to inform decision and actions to be taken during follow-up and counselling. Health facility staff recorded patient's details, surgical procedures and follow-up plan. Post-operative follow-up was routinely done at 1 day, 2 weeks and 3-6 months.

### Data management and analysis

Standardised data collection tool was used. The information was sent to the office of trachoma focal person and entered in excel spreadsheet by health records information officers. The analysis was simple hence it was possible to do it using a spreadsheet. The percentage population coverage was calculated by dividing the number of people enumerated during the pre-survey census by the population projection estimate by the NBS times one hundred. The authors considered the total number of TT cases confirmed in a population or administrative unit to be the total backlog in the unit. The prevalence was computed by dividing the number of cases by the population from which the cases were diagnosed multiplied by 100. The level of achievement of TT elimination threshold was assessed by dividing the number of TT cases diagnosed by total population multiplied by 1,000. The term ''threshold cases'' was used to refer to the number of cases which if operated would reduce the backlog to less than 1 case per 1,000 people while the term ''excess cases''was used to refer to the backlog minus ''threshold cases''. Inter-observer agreement was expressed as a percentage of the observations where a case finder and a TT surgeon (reference examiner) recorded the same diagnosis. An agreement of 80% or higher was set as the minimum requirement for recruitment of case finders in this survey. Where indicated Kappa values were computed using a statistical software and interpreted in line with the Landis and Koch classification [16] as follows: < 0.0 = no agreement, 0.0-0.20 = slight agreement, 0.21-0.40 = fair agreement, 0.41-0.60 = moderate agreement, 0.61-0.80 = substantial agreement and 0.81-1.0 = almost perfect agreement.

### Ethical considerations

This operational research utilised Health Management Information System data provided by Narok County Government and Division of Ophthalmic Services of the Ministry of Health. Details for individual persons are not included. Trachoma project team conducted community mobilisation and took informed verbal consent from community leaders, heads of households and examined individuals. Persons and households which were not willing to be examined/visited during the routine TT screening activities were not forced to do so. The County Government and partners offered free community based surgery for TT and cataract plus treatment for other eye conditions through an existing Primary Eye Care Project. People with complicated eye and medical conditions were treated and/or referred accordingly.

## Results

This operational research resulted in development of the CMMF approach which includes of an innovative TTall survey method anchored on existing community-based surgical services.

### Inter-observer agreement

All cases identified by TT case finders were confirmed to have TT. Therefore, there was 100% agreement on the diagnosis of TT between the case finders and the surgeons. This was attributed to the strict two-stage recruitment criteria for case finders and prolonged training period. The was no indication to calculate Kappa values in this study because all the persons identified to have TT by the case finders were confirmed to be having TT.

### Studied population

Surveys were conducted in 4,784 households in 4 sub-location namely: Esoit, Siana, Megwara and Naikara (Table 2). Siana and Naikara were each divided into 2 because they are expansive. The total projected population was 29,548 and pre-survey census 22,912 people. Coverage was 97.6% of the projected population. There was 101.5% population coverage in Megwara sub-location which the project team attributed to nomadic migrations.

### Backlog and prevalence of TT

A total of 53 cases of TT were diagnosed in a population of 22,912 people in the studied population units. The mean prevalence of TT was 53/22,912 x 100 = 0.23%. This was equivalent to 2.3 cases per thousand population. The project required to operate on at least 30 cases (excess cases) to achieve the elimination threshold of 1 case per 1000 population. The backlog and excess cases for the 4 sub-locations are in Table 2 below.

## Discussion

The CMMF approach will enable Narok to lay claim of having eliminated TT as a public health because the approach reveals all TT cases in the studied areas and the project has capacity to tackle the backlog. The elimination threshold is a dynamic process which requires effective health care systems to identify and treatment new cases to sustain it [1]. This new approach is anticipated to reduce the cost of TT survey because of integration of research into ongoing project activities. The approach also resulted in a shift from a “supply-driven” to a “total-demand-driven” surgical service delivery model which eliminates the need for repeated community mobilization and screening for dwindling TT cases at surgical camps. The TTall survey method used in the new approach had a projected population coverage of 97.6%. This implies that it was likely that 2.4% of the population was missed. Project reports indicated that the nomadic communities in Narok are settling due to ongoing land demarcation where title deeds are issued to individual land owners. The population projections were extrapolated using the 2009 mean inter-censal population growth rate [14] which may not have been applicable in all population units. The growth rate is also likely to change over time. The high inter-observer agreement was attributed to strict recruitment criteria and prolonged apprenticeship period. A study conducted in Narok revealed that lay people can identify [12] which means that this task can be shifted even to unskilled workers. The World Health Organization standard trachoma guidelines do not recommend mandatory validation of TT graders [1, 10, 13]. Certification of elimination of TT as a public health is based on having less than 1 unknown case per 1000 people and availability of systems to manage new cases [1]. Narok project is thus justified to lay claim of having achieved the elimination threshold when all cases are made known using the TTall method. In this study the prevalence of TT in the studied population was 0.23% (2.3 cases per 1000 population) and there were 30 excess TT cases to be operated to achieve the threshold. This implies that the elimination threshold had not been achieved prior to the survey. This new approach requires a relatively settled community since a large population migration can introduce errors calculation of backlog and determination of progress towards elimination. The approach may not be appropriate in settings with high burden of TT where there are adequate cases for the “supply-driven” surgical camps. It was assumed that all the diagnosed TT cases were trachomatous and non-trachomatous TT could not be ruled-out.

## Conclusions

The CMMF approach introduced a paradigm shift from the traditional low productivity “supply driven” TT surgical service delivery model to a more effective “demand driven” model. The total backlog and prevalence of TT in the study area were confirmed using validated community-based case finders. TT elimination threshold had not been achieved.

### What is known about this topic

- There is no standard trachoma prevalence survey method to accurately confirm attainment of the very low elimination of TT threshold of 1 case per thousand population;
- TT is relatively easy to diagnose and even lay people living in the endemic areas in Narok County have local names for it.

### What this study adds

- Utilises non-technical community-based case finders to measure the actual backlog of TT and monitor achievement of elimination threshold as part of ongoing project activities;
- Reduces project cost by eliminating the need for periodic TT impact surveys.

## Competing interests

The authors declare no competing interests.
